# Barriers and facilitators to patient retention in HIV care

**DOI:** 10.1186/s12879-015-0990-0

**Published:** 2015-06-28

**Authors:** Baligh R. Yehia, Leslie Stewart, Florence Momplaisir, Aaloke Mody, Carol W. Holtzman, Lisa M. Jacobs, Janet Hines, Karam Mounzer, Karen Glanz, Joshua P. Metlay, Judy A. Shea

**Affiliations:** Department of Medicine, Perelman School of Medicine at the University of Pennsylvania, Philadelphia, PA USA; Department of Medicine, Drexel University School of Medicine, Philadelphia, PA USA; Department of Pharmacy Practice, Temple University School of Pharmacy, Philadelphia, PA USA; Department of Family Medicine and Community Health, Perelman School of Medicine at the University of Pennsylvania, Philadelphia, PA USA; The Jonathan Lax Center, Philadelphia FIGHT, Philadelphia, PA USA; Department of Biostatistics and Epidemiology, Perelman School of Medicine at the University of Pennsylvania, Philadelphia, PA USA; Department of Biobehavioral Health Sciences, University of Pennsylvania School of Nursing, Philadelphia, PA USA; Division of General Internal Medicine, Massachusetts General Hospital, Boston, MA USA; University of Pennsylvania Perelman School of Medicine, 1021 Blockley Hall, 423 Guardian Drive, Philadelphia, PA 19104 USA

**Keywords:** Retention, Engagement, HIV, Care, Barriers, Facilitators

## Abstract

**Background:**

Retention in HIV care improves survival and reduces the risk of HIV transmission to others. Multiple quantitative studies have described demographic and clinical characteristics associated with retention in HIV care. However, qualitative studies are needed to better understand barriers and facilitators.

**Methods:**

Semi-structured interviews were conducted with 51 HIV-infected individuals, 25 who were retained in care and 26 not retained in care, from 3 urban clinics. Interview data were analyzed for themes using a modified grounded theory approach. Identified themes were compared between the two groups of interest: patients retained in care and those not retained in care.

**Results:**

Overall, participants identified 12 barriers and 5 facilitators to retention in HIV care. On average, retained individuals provided 3 barriers, while persons not retained in care provided 5 barriers. Both groups commonly discussed depression/mental illness, feeling sick, and competing life activities as barriers. In addition, individuals not retained in care commonly reported expensive and unreliable transportation, stigma, and insufficient insurance as barriers. On average, participants in both groups referenced 2 facilitators, including the presence of social support, patient-friendly clinic services (transportation, co-location of services, scheduling/reminders), and positive relationships with providers and clinic staff.

**Conclusions:**

In our study, patients not retained in care faced more barriers, particularly social and structural barriers, than those retained in care. Developing care models where social and financial barriers are addressed, mental health and substance abuse treatment is integrated, and patient-friendly services are offered is important to keeping HIV-infected individuals engaged in care.

## Background

In order to optimally benefit from HIV care and treatment, HIV-infected individuals must complete several steps along a care continuum – HIV testing and diagnosis, linkage to and retention in primary HIV care, and receipt and adherence to antiretroviral therapy (ART) [1, 2]. Retention in care is essential in this process, providing opportunities to monitor response to HIV therapy, prevent HIV-associated complications, and deliver ancillary services [3–9]. Moreover, retention in care improves survival and reduces the risk of HIV transmission to others [10, 11]. Despite these advantages, only 50–75 % of HIV-infected individuals in the United States (U.S.) linked to care meet national retention in care standards (e.g. completion of two or more HIV primary care appointments per year) [2, 12–16].

Multiple cohort and survey studies have examined predictors of retention in care, noting that younger age, male sex, black race/ethnicity, and use of intravenous drugs are associated with poor retention [2, 10, 12–14, 17, 18]. Conversely, patients receiving case management services and individuals with fewer unmet needs are more likely to consistently engage in care than their counterparts [19, 20]. However, these studies are limited by the type of information available in medical records and collected in questionnaires, primarily emphasizing demographic and clinical characteristics. To better understand the full range of factors impacting retention in care, a more qualitative approach is needed.

Some qualitative studies have examined patient-reported barriers and facilitators to retention in care [21–29]. On the patient-level, concerns about privacy, avoidance and disbelief of HIV serostatus, ability to cope with HIV stigma, and substance use have been identified as barriers [21–25]. Patients also described clinic-level barriers such as transportation problems and lack of clinic staff to consistently answer and return phone calls [23, 24, 30]. Facilitators to engagement in care included positive relationships with healthcare providers and a strong social support system [23, 24, 30]. Certain qualitative studies additionally focused on specific populations, such as women of color and those living in rural areas [26–29]. Patient-reported barriers to care in these populations included substance use, depression, stigma, and competing life activities such as family responsibilities and work schedules [26–29]. Similar to other studies, facilitators to care included having a positive patient/provider relationship and access to transportation [26–28].

Our study adds to prior literature by identifying barriers and facilitators to retention in care using contemporary data collected from a diverse population of HIV-infected individuals. Uniquely, we investigate and compare the experiences of patients who are retained and not retained in care to better understand the differences between these groups, including both the number and type of barriers and facilitators to retention in care they report.

## Methods

### Study design, sample, and recruitment strategy

We recruited HIV-infected adults (≥18 years) from three urban, Ryan White Program funded clinics in Philadelphia – Hospital of the University of Pennsylvania MacGregor Infectious Diseases Clinic, Temple University Comprehensive HIV Program, and the Jonathan Lax Treatment Center at Philadelphia FIGHT – between March and November 2013 - to participate in qualitative, semi-structured interviews. Two clinics were university affiliated, and one was community-based. These clinics offered access to many Ryan White-funded and community services including counseling, support groups, transportation assistance, social work services, and case management to help individuals apply for housing and income assistance.

Using purposive sampling, a nonprobability sampling technique whereby subjects are selected because of specific characteristics, patients with varying retention patterns were invited by phone or approached while in the clinic waiting room to participate in the study. All participants provided written informed consent and were compensated $25 for their time. Recruitment concluded when we reached thematic saturation in our sample.

A multidisciplinary research team, comprised of experts in HIV care, health behavior science, and qualitative research methods, was responsible for the study design, data collection, and analysis. The study was approved by the University of Pennsylvania Institutional Review Board, Temple University Institutional Review Board, and Philadelphia FIGHT Institutional Review Board.

### Data collection

For each patient, sociodemographic (age, race/ethnicity, HIV transmission risk factor, health insurance coverage) and clinical data (CD4 cell counts, ART regimen) at the time closest to the interview date were abstracted from the medical record. Retention was based on the U.S. *National HIV/AIDS Strategy* (NHAS) metric, with retained individuals defined as completing 2 or more primary HIV care visits separated by ≥ 90 days in the 12-month period prior to the interview date [31]. Primary HIV care visits refer only to medical care appointments and do not include nursing, pharmacy, laboratory, social services, or other types of visits. HIV viral suppression was categorized as suppressed (HIV-1 RNA ≤ 400 copies/mL) and not suppressed (HIV-1 RNA > 400 copies/mL) based on the median value in the 12-month period before the interview date. A suppression cut-off of 400 copies/mL was chosen to facilitate comparison with other studies.

We developed a semi-structured interview guide designed to elicit patients’ perspectives on managing their HIV infection. Interview questions were based on a literature review of barriers and facilitators to HIV care and treatment, which included 20 articles published over the past 10 years, and discussions with experts involved in the care of people living with HIV. Each interview lasted 20–30 min, and was conducted by a trained interviewer familiar with the study goals and skilled in qualitative interview techniques. The interview began with open-ended questions exploring patients’ experiences with HIV medical care and treatment. Then, patients were asked to reflect on barriers and facilitators to retention in care, relationships with their providers and clinic staff, and experiences navigating the healthcare system. Following this, patients were asked to comment on their health beliefs, support networks, and ability to address problems that may compromise clinic attendance. Finally, patients had the opportunity to share general reflections regarding their experience managing their HIV infection.

After piloting the interview guide with 6 participants, whose data were included in the final analytic sample, the research team met to review early transcripts and adjust the interview guide to better capture patients’ perspectives.

### Data analysis

All interviews were audio recorded, professionally transcribed, and imported into NVivo10 software for analysis (QSR International, Melbourne, Australia). Descriptive analyses of the sample were conducted. Interview data were analyzed for themes and patterns using a modified grounded theory approach, a methodology that involves iterative development of concepts about what is occurring in the data as they are collected [32]. The process develops themes and sub-themes that emerge “from the ground” based on responses to the questions. First, an initial set of transcripts was reviewed line-by-line to generate a working coding scheme. Then, using this scheme, we independently coded a second set of transcripts and revised the scheme until no new themes were identified. A subset of 12 transcripts (approximately 20 % of the total sample) was double-coded to check for inter-rater reliability. Inter-rater reliability was compared across a total of 15 nodes. A kappa could be calculated for 8 of the 15 nodes. Of the 8 nodes where kappa was calculated, the median kappa was 1 with a range of 0.75–1. Of the 7 nodes where kappa could not be calculated, there was perfect agreement for 3 nodes and disagreement in 1 of 12 interview transcripts for 4 nodes. Lastly, a subset of the research team applied the final coding scheme to all transcripts.

After all transcripts were coded, the data was synthesized in summary tables. The frequency for each identified barrier and facilitator to retention in care was categorized into tertiles: high, medium, low. For the total sample and each group of interest (patients retained in care and patients not retained in care), a barrier or facilitator was classified as high if more than 40 % of participants identified the theme, medium if 21–39 % of participants identified the theme, and low if less than 20 % of participants identified the theme. Identified barriers and facilitators were qualitatively compared between the two groups of interest: patients retained in care and those not retained in care.

## Results

A total of 51 HIV-infected patients were interviewed; 25 retained in care and 26 not retained in care. (Table 1) The median age was 45 years (range 24–65), with 27 male patients (53 %) interviewed. Most participants were of minority race/ethnicity (87 %), reported heterosexual transmission as their HIV risk factor (69 %), and were on Medicaid or uninsured (73 %). All but two patients (96 %) were on ART. Over half of the sample (69 %) had a CD4 cell count ≥ 350 cells/mm^3^ and 57 % were virologically suppressed.

**Table 1:** Sample demographic and clinical characteristics, overall and by retention in care status

Characteristic^a^	Total	Retained in care^h^	Not retained in care^h^
	(N = 51)	(N = 25)	(N = 26)
Age (years)^b^			
18-29	4 (8 %)	1 (4 %)	3 (12 %)
30-39	11 (22 %)	2 (8 %)	9 (35 %)
40-49	18 (35 %)	10 (40 %)	8 (31 %)
≥50	18 (35 %)	12 (48 %)	6 (23 %)
Sex			
Male	27 (53 %)	19 (76 %)	8 (31 %)
Female	24 (47 %)	6 (24 %)	18 (69 %)
Race/Ethnicity			
White	6 (12 %)	3 (12 %)	3 (12 %)
Black	41 (80 %)	21 (84 %)	20 (77 %)
Hispanic	3 (6 %)	1 (4 %)	2 (8 %)
Other	1 (2 %)	0 (0 %)	1 (4 %)
HIV risk factor^c^			
MSM	13 (26 %)	8 (32 %)	5 (19 %)
Heterosexual	35 (69 %)	14 (56 %)	21 (81 %)
IDU	3 (6 %)	3 (12 %)	0 (0 %)
Insurance^d^			
Private	4 (8 %)	1 (4 %)	3 (12 %)
Medicaid	34 (67 %)	17 (68 %)	17 (65 %)
Medicare	10 (18 %)	6 (24 %)	4 (15 %)
Uninsured	3 (6 %)	1 (4 %)	2 (8 %)
ART regimen^e^			
PI	29 (57 %)	16 (64 %)	13 (50 %)
NNRTI	15 (29 %)	7 (28 %)	8 (31 %)
Integrase	5 (10 %)	2 (8 %)	3 (12 %)
Not on ART	2 (4 %)	0 (0 %)	2 (8 %)
CD4 cell count (cell/mm^3^)^f^			
≤ 200	8 (16 %)	6 (24 %)	2 (8 %)
201-350	8 (16 %)	2 (8 %)	6 (23 %)
351-500	8 (16 %)	4 (16 %)	4 (15 %)
> 500	27 (53 %)	13 (52 %)	14 (54 %)
Viral suppression^g^			
Suppressed	29 (57 %)	17 (68 %)	12 (46 %)
Not suppressed	22 (43 %)	8 (32 %)	14 (54 %)

*Abbreviations*: *ART* antiretroviral therapy, *MSM* men who have sex with men, *IDU* injection drug use, *PI* protease inhibitor, *NNRTI* non-nucleoside reverse transcriptase inhibitor

^a^Characteristics and values within the 12-month period prior to interview date

^b^Age on the date of interview

^c^Patients who had IDU in combination with another risk factor (e.g. MSM, HET) were classified as IDU

^d^Patients with both Medicare and Medicaid were grouped as Medicare

^e^Patients were considered to be on ART if they concomitantly received ≥ 3 antiretroviral drugs (excluding ritonavir) during the 12-month period prior to the interview date. ART regimen prescribed closest to the interview date was grouped using the following hierarchy: (1) PI-based; (2) NNRTI-based; and (3) integrase inhibitor-based

^f^CD4 cell count closest to the date of interview

^g^HIV viral suppression was categorized as suppressed (HIV-1 RNA ≤ 400 copies/mL) and not suppressed (HIV-1 RNA > 400 copies/mL) based on the median value in the 12-month period before the interview date

^h^Retention in care was defined as completing 2 or more primary HIV care visits separated by ≥ 90 days in the 12-month period prior to the interview date

Overall, participants identified 12 types of barriers and 5 types of facilitators to retention in care. Barriers and facilitators were associated with patient, clinic/health system, and environmental factors. On average, retained individuals endorsed 3 barriers, while persons not retained in care endorsed 5 barriers. Both groups (participants retained and not retained in care) commonly (high tertile) discussed depression and mental illness, feeling sick, and competing life activities as barriers. In addition, individuals not retained in care commonly (high tertile) endorsed expensive and unreliable transportation, experiencing stigma, and insufficient insurance as barriers. On average, participants in both groups referenced 2 facilitators. Both groups commonly (high tertile) discussed the presence of social support, patient-friendly clinic services (transportation, co-location of services, scheduling/reminders), and positive relationships with providers and clinic staff as facilitators.

Tables 2 and 3 categorize barriers and facilitators to retention in care, respectively; display selected quotes; and show the relative frequency (high, medium, low) with which each theme was discussed overall and by each patient group (retained in care and non-retained in care). A more detailed analysis of the barriers and facilitators to retention in care is presented below.

**Table 2:** Barriers to retention in care for people living with HIV

Barriers	Selected patient quotes	Total patients	Retained	Not retained
1. Competing Life Activities	“Of course, mother of four. Work 40-hour full-time job, come home to a full-time job, single mom. So yeah, I mean, there are times, like I said, from exhaustion. Sometimes – most times, it’s just sheer exhaustion. I’m tired, you know. Before I know it, I’m asleep somewhere and I’m sleeping so long, it’s the next day. But when I get up, I will take it. It’s far and few between, but it happens. It happens.” -NR Female	High	High	High
2. Feeling Sick	“Maybe because it was cold or it was raining and I was sick and I didn’t feel like coming, even though I was sick, because I know like in the past I will be feeling sick as hell, but I couldn’t even have the strength to get up to come to see the doctor.” -R Female	High	High	High
3. Stigma	“You don’t want to see nobody you know and all that kind of stuff. I hate the waiting room, I wish I could put on my hood and walk right through there when I leave.”	High	Medium	High
	-R Male			
4. Depression & Mental Illness	“It impacts a lot. It can impact a lot especially depending on I guess my emotional state. Depending on how bad I feel I’m not gonna move at all, I’m just not gonna come out the house, no matter what the reason that may have brought it on, whether it was me, an argument or fight with my husband, the kids driving me crazy today, no matter what brought it on, depending on how bad I feel, I’m just not gonna come because of my depression.” -NR Female	High	High	High
5. Expensive & Unreliable Transportation	“Well, what makes it hard sometimes if you don’t have money to get here. If you don’t have a car, that’s one thing. And what makes it easy is when you have transportation to come here. And it’s accessible if you’re on a bus route. It’s right on the [Specific bus route]. It’s easy to get here. And that’s about it. Sometime you can’t come because you don’t have the money. That’s a factor.” -NR Male	High	Medium	High
6. Insufficient Health Insurance	“I’d say about the last two years, it’s gotten to a point whereas though things they used to cover they don’t cover no more. And being – having this disease, we need a lot of things done. They don’t send out no letter, no nothing, just saying you can’t have this done no more or it’s going to be an extra charge. They don’t say nothing. I get to the place like the dentist. I have bone loss. And I had an appointment. I went to the appointment, and they’re telling me it’s not covered.” -R Male	Medium	Low	High
7. Forgetfulness	“If I’m rushing out of the house and I forget to just grab them and put them in my purse or something. That’s most of the time when I forget.” -R Female	Medium	Medium	Medium
8. Substance Abuse	“I forgot a lot of appointments. I was on drugs and I didn’t – I wouldn’t come in for like months, six months, to a year. I’m just getting back on track.” -R Female	Medium	Medium	Medium
9. Negative Experiences with Clinic Spaces & Processes	“I still went…He just run in and out of the office, leave me sitting – first of all, you sat an hour just to get triaged. Then they stuck in a room, you sat another half an hour, 45 min. Then he’d come and he’s be on the phone, he’d be in and out, just write you a script and send you on your way. Every once in a while he gave you blood work. Back then it was like an easy gig. But I left him and I found – the Gods called up, no for real man, it was a blessing.” -R Male	Medium	Medium	Medium
10. Challenges with Appointment Scheduling	“Then if I come to appointment, I have to schedule back and forth. She say 11:30, but on the paper I got last time, it was 12:30. So I get her 12:00, she say it’s too late, I have to go back and reschedule. And I rescheduled again for the 26th, it took another month.” -NR Female	Low	Low	Medium
11. Difficult Relationships with Clinic Staff Including Providers	“The social worker, [Participant’s Social Worker], and I am not a fan of her. I am not a fan of her. She thinks she’s here doing you a favor. She is manipulative, ring the rule, send you up the steps and down and around and about and then all around. I cannot take that social worker behavior. Just get to the point and lead me in the right direction and tell me what the steps to take. I’m not here to ask you for a handout or what you can offer me personally from a clinical standpoint, a professional standpoint, please assist me with this matter. She make it seem as if she is doing you a favor.” -R Female	Low	Low	Medium
12. Inconsistent, Unstable, or Inadequate Housing	“I’m going through a situation right now with my living conditions. I haven’t took my medications in about three weeks now. I discussed - because I am going through – I’m living right now in a warehouse with my cousin who was also evicted because of some of the legal bullcrap we had to go through with my niece and other stuff which annoys me. But right now I’m staying with him so I’m going through a lot of stress with that. I guess I could have continued taking it but I just never been down to get the pills or whatnot.” -R Male	Low	Low	Low

*Abbreviations*: *R* retained, *NR* not retained

The frequency for each identified barrier to retention in care was categorized into tertiles: high, medium, low

**Table 3:** Facilitators to retention in care for people living with HIV

Facilitator	Selected patient quotes	Total patients	Retained	Not retained
1. Positive Relationships with Clinic Staff Including Provider	“When Dr. [Doctor’s Name] speaks, I take it to heart because I know he is really concerned about me. I don’t know about every other doctor. I can only tell you about Dr. [Doctor’s Name]. I know he’s concerned and I know his concerns are valid so when he suggests or says, okay, [Name], I always try to do it.” -R Male	High	High	High
2. Social Support	“Sometimes I’m not able to go to the food bank, but I’ll call my children. I’ve called my two daughters or my son, and somebody will drop something off. They say, ma, we don’t want you to be there and not have nothing to eat. We know it’s important that you’ve got to take your medicines. So sometimes when I can’t make it to the food bank, they look out for me and bring me something over to the house.” -R Female	High	High	High
3. Patient-friendly Clinic Services	“Because they give you transportation back and forth… So they make sure I have that when I come. So there’s really no excuse.” -R Female	High	High	High
	“They usually call me the day before or a couple days before and I usually put it in my phone on my calendar.” -NR Male			
	“So it’s just convenient that everything is in one place, I can go to the doctors, I can get my medicine, I can go to my groups, and I can do this all in one, between the two buildings.” -NR Female			
4. Patient Initiated Reminder Strategies	I: “What things help you making your appointments?”	Low	Low	Medium
	R: “The alarm on my phone. It’s aggravating. I’ll turn it off, it’ll turn back on… Yeah. I fixed it that way. I got five alarms and I’ll set them all 10 min apart.” -R Male			
5. Flexible Schedule	“Basically just me. I just will go. I mean I try to schedule where I don’t have nothing to do that week. When nothing else is coming up and if I have an appointment, it will just be my appointment that I have to go to that week without anything else bothering me. Clearing it out, yeah.” -R Female	Low	Low	Low

*Abbreviations*: *R* retained, *NR* not retained

The frequency for each identified facilitator to retention in care was categorized into tertiles: high, medium, low

### Barriers to retention in care

#### Competing life activities

Regular attendance at clinic is not always the top priority for some participants; competing life activities was a barrier in the high tertile for both those individuals retained and not retained in care. Caring for children or elderly family members, work, and school were among the most commonly mentioned obstacles. Requesting time off for appointments was difficult and some participants mentioned struggling to find a job that was flexible enough to allow them to effectively manage their HIV infection.

#### Feeling sick

Feeling sick was a barrier in the top tertile for both groups and was commonly a reason for skipping or rescheduling appointments. Participants related their symptoms to a range of factors including medication side effects, compromised immune systems that made common colds and the flu more potent, and feeling emotionally low.

#### Stigma

Stigma was a barrier in the high tertile for patients not retained in care and a barrier in the medium tertile for those retained in care. Many participants reported hesitancy to disclose their status to family, friends, and acquaintances. Uncertainty about how family, friends, or the public would respond to their status made some patients anxious and affected their ability to attend appointments. Attempting to avoid disclosure in the waiting room, laboratory, and pharmacy created additional obstacles for these participants and discouraged regular clinic attendance.

#### Depression and mental illness

Participants in both groups commonly (high tertile) identified symptoms of depression and other mental illnesses (e.g. post-traumatic stress disorder, schizophrenia, anxiety, and bipolar disorder) as barriers. Participants experiencing depression described sleeping through appointments and sometimes not wanting to “bother” with traveling to clinic. In addition, depressed participants felt apathetic about their ’health care, with some stating that they did not care whether they lived or died.

#### Expensive and unreliable transportation

Patients who were not retained in care more often (high tertile) discussed transportation-related challenges relative to other barriers, as compared to retained patients where transportation-related challenges were in the medium tertile. Specific issues included the inability to afford bus/subway passes or carfare, unreliable shuttle van services, and the impact of inclement weather on public transportation and bike riding. Some participants also mentioned heavy traffic and the cost and availability of parking as barriers.

#### Insufficient health insurance

Non-retained patients more commonly (high tertile) expressed challenges with health insurance as compared to retained patients (low tertile). Participants found the process of enrolling in health insurance complicated and slow, affecting their ability to schedule appointments and receive medications in a timely manner. In addition, co-pays associated with medical visits deterred some participants from seeking care.

#### Forgetfulness

Both groups sometimes (medium tertile) discussed challenges remembering appointment dates. Losing an appointment slip, not writing a reminder note, or not entering information into a phone or calendar were common actions that led to missed appointments. Participants also referenced having a busy lifestyle as an obstacle to remembering appointment dates.

#### Substance abuse

Substance abuse was in the medium tertile for both patients retained and not retained in care. These participants described forgetting or dismissing thoughts about attending appointments when actively using drugs. Retained participants mentioned substance abuse as an issue they struggled with in the past but had overcome.

#### Negative experiences with clinic space and processes

Participants in both groups sometimes (medium tertile) referenced negative experiences at their clinic. Several participants mentioned frustration about clinic wait times that can extend to several hours; some also mentioned frustration that being 15 min late could result in a cancelled appointment. Many participants disliked the waiting room experience for a range of reasons including fear of unintentional disclosures and conflict with other patients. Challenges with referral paperwork and long waits for laboratory testing were also mentioned as barriers to appointment adherence.

#### Challenges with appointment scheduling

Non-retained patients more often (medium tertile) discussed difficulties with scheduling appointments as compared to other barriers, while this barrier was in the lowest tertile for retained participants. Patients described challenges with their clinic’s scheduling system and limited hours as well as their own lack of privacy when scheduling appointments over the phone.

#### Difficult relationships with clinic staff including providers

Both groups mentioned strained relationships with current or past health care providers or clinic staff; though this barrier was in the middle tertile for non-retained patients and in the low tertile for retained patients. Participants were sometimes unwilling to share details about their health or listen to providers’ instructions if they felt patronized or disrespected.

#### Inconsistent, unstable, or inadequate housing

Unstable housing was a barrier mentioned (low tertile) by both patients retained and not retained in care. For some participants, housing insecurity caused significant stress and created obstacles for daily living that affected appointment adherence. Without a stable phone number, participants were unable to receive reminder phone calls and maintain contact with their provider. Also without a place to shower and bathe, participants were self-conscious about visiting the clinic. In addition, frequent address changes sometimes prevented patients from completing the necessary paperwork required to maintain their health insurance.

### Facilitators of retention in care

#### Positive relationships with clinic staff including providers

For both the retained and non-retained groups, positive relationships with clinic staff including the HIV providers was one of the most commonly (high tertile) discussed facilitators. Patients reported that a strong relationship with their provider increased confidence in their provider’s recommendations and advice. Moreover, having a supportive patient-provider relationship created a sense of trust, allowing patients to honestly share their health experiences and adherence behaviors. Many patients stated that they enjoyed coming to the clinic and felt supported by clinic staff. These patients described staff as professional, sincere, patient, and caring.

#### Social support

Both groups commonly (high tertile) referenced people in their lives who supported their appointment adherence. Children, siblings, partners, relatives, friends, neighbors, clinic staff, and support groups were all discussed as important sources of support. These people and groups reminded and motivated participants to attend appointments, and helped assure that participants had food and transportation.

#### Patient-friendly clinic services

Patients retained and not retained in care commonly (high tertile) discussed a range of clinic services that made attending appointments easier, including transportation assistance, convenient scheduling processes, reminder phone calls, and co-location of medical and ancillary care services. Non-retained and retained patients both commonly referenced the benefit of transportation services, including free bus/subway passes and van services. Similarly, both groups mentioned the value of a convenient scheduling process, which included the ability to schedule appointments over the phone or in-person; talking to a live person instead of an automated system; and schedulers that work with patients to find the earliest and most convenient appointment time. Participants also discussed the benefit of reminder phone calls in facilitating appointment adherence. Some participants appreciated the ability to accomplish multiple tasks in the same location, such as participating in a research study or attending a support group before or after a scheduled appointment.

#### Patient initiated reminder strategies

The benefits of appointment reminder tools were referenced by both retained and non-retained patients; however, this facilitator was in the medium tertile for those non-retained in care and in the low tertile for those retained in care. Participants commonly mentioned personal systems like calendars and alarms for organizing appointment schedules. Participants inputted appointment date information into electronic (mobile device) and paper calendars and set alarms to remind themselves of upcoming appointments.

#### Flexible schedule

An open, flexible schedule was identified by some (low tertile) retained and not retained patients as a facilitator of retention in care. The ability to schedule appointments at multiple times during the day made it easier to get and keep appointments. Clearing a schedule on appointment days or even during the week of an appointment was mentioned as a strategy for assuring appointment attendance.

## Discussion

Retention in care is a critical element of the HIV care continuum and is necessary for successfully managing HIV infection. This study adds to the existing literature by examining differences in barriers and facilitators to retention in care for patients with varying retention patterns. Individuals in the retained and non-retained groups expressed common barriers and facilitators to retention in care. However, as a group, non-retained individuals identified more barriers and more often discussed stigma, expensive and unreliable transportation, insufficient health insurance, challenges with appointment scheduling, and difficult relationships with clinic staff as obstacles.

Participants from both groups described common struggles to consistently attending clinic visits, including dealing with competing life priorities (e.g. caring for children or elderly family members), feeling physically sick, and being depressed. Caregivers of chronically ill individuals, particularly in underprivileged populations, may experience substantial economic strains due to lost wages, social isolation, and depressive symptoms [33–35]. Moreover, studies have shown that caregiver responsibilities may prevent people from attending their own appointments or reaching their own full health potential [36]. The use of new technologies, including secure electronic messaging and videoconferencing, could address some of these barriers by increasing access to care and medical information [37, 38]. However, these modalities have been limited by lack of uptake and integration with our current financial reimbursement systems, privacy concerns, and provider comfort using these technologies [39]. Consistent with other studies, patients who felt sick or depressed were more likely to miss their appointments [28, 29, 36, 40–42]. Successfully integrating psychiatric and psychosocial treatment into HIV care, when possible, may serve as a tool for improving both retention in care and HIV clinical outcomes [43–46].

Participants in the non-retained group more commonly identified stigma, expensive and unreliable transportation, insufficient health insurance, challenges with appointment scheduling, and difficult relationships with clinic staff as barriers to retention in care compared to the retained group. While major advances in the treatment of HIV have been made, negative perceptions and stigma associated with the disease have not evolved as rapidly [47, 48]. Non-retained individuals commonly (high tertile) cited stigma as a barrier compared to retained individuals (middle tertile). This difference may be a consequence of different experiences or varying perceptions of similar experiences between the groups. Patients retained in care may also have stronger social supports or access to mental health care, which have been identified as protective against stigma [49, 50], than those not retained in care. Additional studies are needed to better understand how patients perceive stigma and its subsequent impact on health behaviors, particularly among individuals with otherwise similar social, economic, and behavior backgrounds and experiences. Interventions, such as skill building through peer coaching, education programs to gain a better understanding of HIV disease, and connecting HIV-infected individuals with community resources and peers, may help patients combat stigma and improve their engagement in healthcare [19, 51, 52].

Expensive and unreliable transportation was commonly (high tertile) discussed as a barrier in the non-retained group, but was in the middle tertile for the retained group. Differences in income, place of residence, and access to individuals who can provide transportation may explain this finding. Prior studies demonstrate that clinics providing support services, including transportation and case management, have better retention rates than those without these services [36, 53–55]. Insufficient health insurance was a high tertile barrier for not retained patients and a low tertile barrier for retained individuals, despite both groups having a similar insurance distribution. While both groups had similar insurance patterns, it may be that those retained in care are better able to navigate the healthcare system and use their insurance coverage effectively to obtain care than those not retained.

Challenges with appointment scheduling and difficult relationships with clinic staff were both in the lowest tertile of barriers for the retained group, but in the middle tertile for patients not retained in care. Satisfaction with the clinic experience predicts whether or not patients return for care [56, 57]. Moreover, patients’ perception of the clinic experience depends not only on the quality of clinical care delivered but also on interactions with clinic staff, appointment wait times, and scheduling efficiency [56, 57]. Among people living with HIV, satisfaction with care has been shown to be positively associated with retention in care and adherence to ART [58]. Non-retained patients may have been less satisfied with their clinic experience and for that reason did not return for appointments or remain engaged in care. Additional research is needed to better understand the differences between patients retained and not retained in care, since despite similar demographic characteristics between the groups they differed in their perceptions about barriers to care.

Both the retained and non-retained groups commonly (high tertile) mentioned supportive patient-provider/patient-staff relationships, patient-friendly clinic services, and social support as highly important facilitators to retention in care. Studies examining the patient-provider relationship have found that interaction styles that reduce social distance with the patient and improve patient comprehension of health issues lead to improved engagement in HIV care [59, 60]. Similarly, patient-friendly clinic services, such as patient orientation to the clinic or open access scheduling, have been documented to reduce missed appointments [36]. In HIV infection, brief face-to-face meetings with clinic staff upon returning for care, interim visit calls, appointment reminder calls, and missed visit follow-up calls improved visit adherence in a randomized control trial of usual care versus enhanced personal contact [61].

There are several limitations to this study. Though we were able to recruit a large number of individuals not retained in care, there may be differences in those patients who were unable to be recruited. These patients may be even less engaged in care, and thus may have different barriers and facilitators than participants in this study. Additionally, patients’ responses may have been influenced by social desirability bias. Ensuring confidentiality and training interviewers to avoid judgmental reactions helped minimize this risk. Finally, the findings of this study may not generalize to other populations, as our patients, clinical practices, and geographic and cultural environment may vary from others. Moreover, not all barriers and facilitators identified may apply to the same degree across populations and locales.

## Conclusions

This qualitative analysis builds on prior research, which describe barriers and facilitators to engagement in care, by comparing the type and frequency of barriers and facilitators between individuals retained and not retained in care. This analysis offers insights for providers, clinic administrators, and health policy makers seeking to improve retention in care. Developing care models where social and financial barriers are routinely assessed and addressed, mental health and substance abuse treatment is integrated, and patient-friendly services are offered is important for keeping HIV-infected individuals engaged in care and for meeting national retention metrics.

## References

[1] Centers for Disease Control and Prevention (CDC) (2011). Vital signs: HIV prevention through care and treatment–United States. MMWR Morb Mortal Wkly Rep.

[2] Fleishman JA, Yehia BR, Moore RD, Korthuis PT, Gebo KA (2012). Establishment, retention, and loss to follow-up in outpatient HIV care. J Acquir Immune Defic Syndr.

[3] Mugavero MJ (2008). Improving engagement in HIV care: what can we do?. Top HIV Med.

[4] Ulett KB, Willig JH, Lin HY (2009). The therapeutic implications of timely linkage and early retention in HIV care. AIDS Patient Care STDs.

[5] Berg MB, Safren SA, Mimiaga MJ, Grasso C, Boswell S, Mayer KH (2005). Nonadherence to medical appointments is associated with increased plasma HIV RNA and decreased CD4 cell counts in a community-based HIV primary care clinic. AIDS Care.

[6] Giordano TP, White AC, Sajja P (2003). Factors associated with the use of highly active antiretroviral therapy in patients newly entering care in an urban clinic. J Acquir Immune Defic Syndr.

[7] Lucas GM, Chaisson RE, Moore RD (1999). Highly active antiretroviral therapy in a large urban clinic: risk factors for virologic failure and adverse drug reactions. Ann Intern Med.

[8] Yehia BR, Kangovi S, Frank I (2013). Patients in transition: avoiding detours on the road to HIV treatment success. AIDS.

[9] Yehia BR, Fleishman JA, Moore RD, Gebo KA. Retention in Care and Health Outcomes of Transgender Persons Living With HIV. Clin Infect Dis. 2013;57(5):774-6. 10.1093/cid/cit363 PMC3739465 23723203

[10] Mugavero MJ, Lin HY, Willig JH (2009). Missed visits and mortality among patients establishing initial outpatient HIV treatment. Clin Infect Dis.

[11] Metsch LR, Pereyra M, Messinger S (2008). HIV transmission risk behaviors among HIV-infected persons who are successfully linked to care. Clin Infect Dis.

[12] Yehia BR, Fleishman JA, Metlay JP (2012). Comparing different measures of retention in outpatient HIV care. AIDS.

[13] Rebeiro P, Althoff KN, Buchacz K (2013). Retention among North American HIV-infected persons in clinical care, 2000-2008. J Acquir Immune Defic Syndr.

[14] Mugavero MJ, Westfall AO, Zinski A (2012). Measuring retention in HIV care: the elusive gold standard. J Acquir Immune Defic Syndr.

[15] HI Hall ELF, P Rhodes, DR Holtgrave, C Furlow-Parmley, T Tang, KM Gray, SM Cohen, J Skarbinsk. Continuum of HIV care: differences in care and treatment by sex and race/ethnicity in the United States. Paper presented at: AIDS 2012; Washington, DC.

[16] Mugavero MJ, Amico KR, Horn T, Thompson MA (2013). The state of engagement in HIV care in the United States: from cascade to continuum to control. Top HIV Med.

[17] Giordano TP, Visnegarwala F, White AC (2005). Patients referred to an urban HIV clinic frequently fail to establish care: factors predicting failure. AIDS Care.

[18] Giordano TP, Hartman C, Gifford AL, Backus LI, Morgan RO (2009). Predictors of retention in HIV care among a national cohort of US veterans. HIV Clin Trials.

[19] Cunningham CO, Sohler NL, Wong MD (2007). Utilization of health care services in hard-to-reach marginalized HIV-infected individuals. AIDS Patient Care STDs.

[20] Rumptz MH, Tobias C, Rajabiun S (2007). Factors associated with engaging socially marginalized HIV-positive persons in primary care. AIDS Patient Care STDs.

[21] Beer L, Fagan JL, Valverde E, Bertolli J (2009). Never in Care P. Health-related beliefs and decisions about accessing HIV medical care among HIV-infected persons who are not receiving care. AIDS Patient Care STDs.

[22] Konkle-Parker DJ, Amico KR, Henderson HM (2011). Barriers and facilitators to engagement in HIV clinical care in the Deep South: results from semi-structured patient interviews. J Assoc Nurses AIDS Care.

[23] Rajabiun S, Mallinson RK, McCoy K (2007). “Getting me back on track”: the role of outreach interventions in engaging and retaining people living with HIV/AIDS in medical care. AIDS Patient Care STDs.

[24] Mallinson RK, Relf MV, Dekker D, Dolan K, Darcy A, Ford A (2005). Maintaining normalcy: a grounded theory of engaging in HIV-oriented primary medical care. Adv Nurs Sci.

[25] Smith LR, Fisher JD, Cunningham CO, Amico KR (2012). Understanding the behavioral determinants of retention in HIV care: a qualitative evaluation of a situated information, motivation, behavioral skills model of care initiation and maintenance. AIDS Patient Care STDs.

[26] Kempf MC, McLeod J, Boehme AK (2010). A qualitative study of the barriers and facilitators to retention-in-care among HIV-positive women in the rural southeastern United States: implications for targeted interventions. AIDS Patient Care STDs.

[27] Quinlivan EB, Messer LC, Adimora AA (2013). Experiences with HIV testing, entry, and engagement in care by HIV-infected women of color, and the need for autonomy, competency, and relatedness. AIDS Patient Care STDs.

[28] Messer LC, Quinlivan EB, Parnell H (2013). Barriers and facilitators to testing, treatment entry, and engagement in care by HIV-positive women of color. AIDS Patient Care STDs.

[29] Moneyham L, McLeod J, Boehme A (2010). Perceived barriers to HIV care among HIV-infected women in the Deep South. J Assoc Nurses AIDS Care.

[30] Christopoulos KA, Massey AD, Lopez AM (2013). “Taking a half day at a time”: patient perspectives and the HIV engagement in care continuum. AIDS Patient Care STDs.

[31] The White House Office of National HIV/AIDS Policy. The National HIV/AIDS Strategy of the United States. 2010.

[32] Tashakkori ATC (2010). Handbook of mixed methods in social and behavioral research.

[33] Mitchell MM, Knowlton A (2012). Caregiver role overload and network support in a sample of predominantly low-income, African-American caregivers of persons living with HIV/AIDS: a structural equation modeling analysis. AIDS Behav.

[34] Emanuel EJ, Fairclough DL, Slutsman J, Emanuel LL (2000). Understanding economic and other burdens of terminal illness: the experience of patients and their caregivers. Ann Inten Med.

[35] Grunfeld E, Coyle D, Whelan T (2004). Family caregiver burden: results of a longitudinal study of breast cancer patients and their principal caregivers. Can Med Assoc J.

[36] Horstmann E, Brown J, Islam F, Buck J, Agins BD (2010). Retaining HIV-infected patients in care: where are we? Where do we go from here?. Clin Infect Dis.

[37] Hersh WR, Helfand M, Wallace J (2001). Clinical outcomes resulting from telemedicine interventions: a systematic review. BMC Med Inform Decis Mak.

[38] Charles BL (2000). Telemedicine can lower costs and improve access. Healthc Financ Manag.

[39] Dixon RF (2010). Enhancing primary care through online communication. Health Aff.

[40] Weiser SD, Riley ED, Ragland K, Hammer G, Clark R, Bangsberg DR (2006). BRIEF REPORT: factors associated with depression among homeless and marginally housed HIV-infected men in San Francisco. J Gen Intern Med.

[41] Riley ED, Wu AW, Perry S (2003). Depression and drug use impact health status among marginally housed HIV-infected individuals. AIDS Patient Care STDs.

[42] Tello MA, Jenckes M, Gaver J, Anderson JR, Moore RD, Chander G (2010). Barriers to recommended gynecologic care in an urban United States HIV clinic. J Womens Health.

[43] Wyatt GE, Longshore D, Chin D (2004). The efficacy of an integrated risk reduction intervention for HIV-positive women with child sexual abuse histories. AIDS Behav.

[44] Weber R, Christen L, Christen S (2004). Effect of individual cognitive behaviour intervention on adherence to antiretroviral therapy: prospective randomized trial. Antivir Ther.

[45] Safren S, Knauz R, O’Cleirigh C (2006). CBT for HIV medication adherence and depression: process and outcome at post-treatment and three-month cross over. Ann Behav Med.

[46] Sikkema KJ, Wilson PA, Hansen NB (2008). Effects of a coping intervention on transmission risk behavior among people living with HIV/AIDS and a history of childhood sexual abuse. J Acquir Immune Defic Syndr.

[47] Herek GM, Capitanio JP, Widaman KF (2002). HIV-related stigma and knowledge in the United States: prevalence and trends, 1991-1999. Am J Public Health.

[48] Grov C, Golub SA, Parsons JT, Brennan M, Karpiak SE (2010). Loneliness and HIV-related stigma explain depression among older HIV-positive adults. AIDS Care.

[49] Logie C, Gadalla TM (2009). Meta-analysis of health and demographic correlates of stigma towards people living with HIV. AIDS Care.

[50] Emlet CA, Brennan DJ, Brennenstuhl S (2013). Protective and risk factors associated with stigma in a population of older adults living with HIV in Ontario, Canada. AIDS Care.

[51] Bradford JB (2007). The promise of outreach for engaging and retaining out-of-care persons in HIV medical care. AIDS Patient Care STDs.

[52] Stangl AL, Lloyd JK, Brady LM, Holland CE, Baral S (2013). A systematic review of interventions to reduce HIV-related stigma and discrimination from 2002 to 2013: how far have we come?. J Int AIDS Soc.

[53] Keller SC, Yehia BR, Momplaisir FO, Eberhart MG, Share A, Brady KA (2014). Assessing the overall quality of health care in persons living with HIV in an Urban environment. AIDS Patient Care STDs.

[54] Higa DH, Marks G, Crepaz N, Liau A, Lyles CM (2012). Interventions to improve retention in HIV primary care: a systematic review of US studies. Curr HIV/AIDS Rep.

[55] Gardner EM, McLees MP, Steiner JF, del Rio C, Burman WJ (2011). The spectrum of engagement in HIV care and its relevance to test-and-treat strategies for prevention of HIV infection. Clin Infect Dis.

[56] Billing K, Newland H, Selva D (2007). Improving patient satisfaction through information provision. Clin Experiment Ophthalmol.

[57] Salisbury C, Wallace M, Montgomery AA (2010). Patients’ experience and satisfaction in primary care: secondary analysis using multilevel modelling. BMJ.

[58] Dang BN, Westbrook RA, Black WC, Rodriguez-Barradas MC, Giordano TP (2013). Examining the link between patient satisfaction and adherence to HIV care: a structural equation model. PLoS ONE.

[59] Mallinson RK, Rajabiun S, Coleman S (2007). The provider role in client engagement in HIV care. AIDS Patient Care STDs.

[60] McCoy L (2005). HIV-positive patients and the doctor-patient relationship: perspectives from the margins. Qual Health Res.

[61] Gardner LI, Giordano TP, Marks G (2014). Enhanced personal contact with HIV patients improves retention in primary care: a randomized trial in 6 US HIV clinics. Top HIV Med.

